# 

**Published:** 1875-08

**Authors:** 


[Vol. XV] AUGUST, 1875. [No. 1.]

### BUFFALO

### Medical and Surgical Journal.

EDITED BY JULIUS F. MINER, M. D.

Professor of Special Surgery in the Buffalo Medical College; Surgeon to the Buffalo General Hospital; Surgeon to Buffalo Hospital of the Sisters of Charity, etc., etc.

AND

EDWARD N. BRUSH, M. D.

BUFFALO.

PUBLISHED FOR THE EDITORS.

1875.

